# Genetic variation in chicken interferon signalling pathway genes in research lines showing differential viral resistance

**DOI:** 10.1111/age.13233

**Published:** 2022-06-23

**Authors:** Joshua Mountford, Almas Gheyas, Lonneke Vervelde, Jacqueline Smith

**Affiliations:** ^1^ The Roslin Institute and Royal (Dick) School of Veterinary Studies University of Edinburgh Midlothian UK

**Keywords:** chicken, disease resistance, genetic variation, inbred lines, interferon

## Abstract

Avian viruses of economic interest are a significant burden on the poultry industry, affecting production traits and resulting in mortality. Furthermore, the zoonosis of avian viruses risks pandemics developing in humans. Vaccination is the most common method of controlling viruses; however current vaccines often lack cross‐protection against multiple strains of each virus. The mutagenicity of these viruses has also led to virulent strains emerging that can overcome the protection offered by vaccines. Breeding chickens with a more robust innate immune response may help in tackling current and emerging viruses. Understanding the genetic evolution of different lines will thus provide a useful tool in helping the host in the fight against pathogens. This study focuses on the interferon genes and their receptors in different chicken lines that are known to be more resistant or susceptible to particular avian viruses. Comparing genotypic differences in these core immune genes between the chicken lines may explain the phenotypic differences observed and aid the identification of causative variations. The relative resistance/susceptibility of each line to viruses of interest (Marek’s disease virus, infectious bursal disease, infectious bronchitis virus and avian influenza virus) has previously been determined. Here we identify single nucleotide polymorphisms in interferons and downstream genes. Functional prediction tools were used to identify variants that may be affecting protein structure, mRNA secondary structure or transcription factor and micro‐RNA binding sites. These variants were then considered in the context of the research lines and their distribution between phenotypes. We highlight 60 variants of interest in the interferon pathway genes that may account for susceptibility/resistance to viral pathogens.

## INTRODUCTION

**FIGURE 1:**
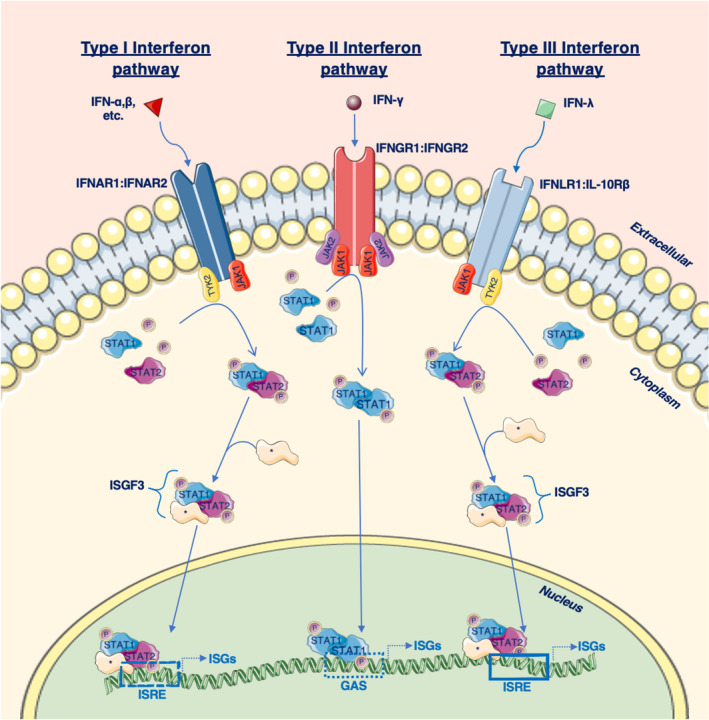
The current model for the interferon response pathway of the chicken. Type I, II and III interferon molecules act as cytokines in a paracrine and autocrine fashion by interacting with the IFNAR1:IFNAR2, IFNGR1:IFNGR2 and IFNLR1:IL‐10Rβ dimeric receptor complexes respectively. Upon receptor binding, the Janus kinase (JAK) signal transducer and activation of transcription (STAT) pathway is activated, whereby phosphorylation of STAT1 and STAT2 causes them to dimerise. In mammals this dimer then goes on to form the complex interferon stimulated gene factor 3 (ISGF3 – in mammals this complex forms via interactions with IRF9, however a chicken orthologue of this protein has yet to be identified). The ISGF3 complex can then enter the nucleus and bind to interferon stimulated response element sequences, resulting in the upregulation of hundreds of ISGs and a specific antiviral state. In the case of the type II system, STAT1 and STAT2 form a homodimer that can then enter the nucleus and bind to gamma‐activated sequences, resulting in upregulation of specific ISGs (Andreakos et al., 2019; Santhakumar et al., 2017b). This image was created using modified images from Servier Medical Art, licensed under a Creative Common Attribution 3.0 Generic License (http://smart.servier.com)

Study of the innate immune system is important as it is seen as a generalised first line of defence and a step in the initiation of immune responses to a pathogen. Induction of the interferon (IFN) signalling pathway is central to this response. The IFN system in chickens is of particular interest as it bears many similarities to the human system, but its simplified nature makes it an ideal model for studying this system. The interferon response in chickens is induced by pattern recognition receptors (PRRs) that react to pathogenic materials such as viral RNA, and this causes a signal cascade, resulting in upregulation of the type I (*IFNα*, *IFNβ*, *IFNκ*), type II (*IFNγ*) and type III (*IFNλ*) interferon genes. Once produced, these interferons can induce an antiviral state by interacting with complementary receptor complexes as detailed in Figure 1 (Santhakumar et al., 2017a, Santhakumar et al., 2017b).

In vertebrate species, it was initially believed that type I and III interferons were redundant owing to the similarity in signal cascade and interferon stimulated genes (ISGs) affected. However, whilst type I receptors are expressed in most cells, the *IL‐28Rα* (*IFNLR1*) receptor appears to be preferentially expressed in specific cells such as epithelial cells. This suggests that the type I system works in a more systemic fashion compared with the cell‐specific type III interferon system (Andreakos et al., 2019; Santhakumar et al., 2017b). Furthermore, inducing the expression of *IFNLR1* in chicken fibroblasts allows IFNλ to induce the type III antiviral response, further supporting the view that the distribution of this receptor determines the antiviral response in these cells (Reuter et al., 2014). The type II IFN system in vertebrate species is unique in comparison as its function is predominantly restricted to immune cells and IFNγ is principally produced by natural killer cells (Lee & Ashkar, 2018). Despite the overlap between the systems highlighted in Figure 1, it is evident the interferon systems play a distinct role in vertebrate species.

The economic impact of avian viruses on the poultry industry cannot be overstated. Infections can result in a reduction in egg production, the condemnation of consumable meat, immunosuppression leading to secondary infections and mortality. In the context of this study, we will focus on resistance to four economically important viruses: Marek’s disease virus (MDV) (Boodhoo et al., 2016), infectious bursal disease virus (IBDV) (Dey et al., 2019), infectious bronchitis virus (IBV) (Legnardi et al., 2020) and avian influenza virus (AIV), which also poses a potential threat to human health (Lycett et al., 2019). Attempts to control these viruses by vaccination or alleviate the impacts of disease have been utilised over the years with varying degrees of success. Alongside the improvement of vaccines and/or vaccine adjuvants the identification of disease resistance genes can feed into conventional breeding programmes, but the selection of birds with improved innate immune responsiveness may result in a broader effect than the selection of those with resistance to specific diseases (Kaiser, 2010; Swaggerty et al., 2009). Studies from MHC‐congenic lines indicate that there are MHC‐related and non‐MHC related factors involved in viral disease resistance. Research using inbred/partially inbred chicken lines in response to viral disease has identified transcriptomic variations either in the interferon genes or in their up‐ or downstream effectors (Kaufman, 2018; Smith et al., 2011; Smith et al., 2015a; Stone, 1975). Given their role in the innate immune response and their variability in expression in response to viruses, it is possible that genetic variants in the interferon pathways between different chicken lines may contribute to resistant and susceptible phenotypes. Identifying variants causative for susceptibility or resistance could therefore allow for the development of breeding schemes to introduce viral resistance into commercial lines and also increase knowledge of virus–host interactions.

The goal of the current *in silico* study was to identify genetic variants from whole genome sequence data from eight chicken research lines and to annotate these variants, i.e. to determine if they are exonic, intronic, upstream or downstream of a gene. Functional prediction tools were used to determine the impact of these variants. Finally, using prior knowledge of the lines’ susceptibility and resistance to IBV, IBDV, AIV and MDV, variants have been correlated to the disease response phenotype with the aim of identifying mutations potentially responsible for specific resistance or susceptibility.

## MATERIALS AND METHODS

### Determination of chicken line phenotype

The research lines examined in this study were lines WI, 15I, 7_2_, 6_1_, C.B12, N, 0 and P2a which have been previously described (Bacon et al., 2000; Mohd Isa et al., 2020; Stone, 1975). These are all White Leghorn lines inbred to varying degrees. The level of inbreeding is indicated in Table S1. Lines 6_1_, 7_2_, 15I and C are highly inbred (with inbreeding coefficients ranging from 0.72 to 0.85), whereas lines N, P2a and Wl are only partially inbred (with inbreeding coefficients ranging from 0.37 to 0.59). Line 0, so called because it contains no avian leukosis virus subgroup E (ALVE) genes, was not developed as an inbred line, but does show a low level in this study. Data from Kaiser et al. (2008) was considered as a starting point for identifying line resistance and susceptibility phenotypes. However, these data were generated in the 1990s and contradictory findings have since been identified (Farhanah et al., 2018; Smith et al., 2015b). A broad search of the literature was completed in order to classify lines as either susceptible or resistant to each virus, as much as was possible. Studies of IBV (Bumstead et al., 1989; Cook et al., 1990; Nakamura et al., 1991; Otsuki et al., 1990), IBDV (Asfor et al., 2021; Bumstead et al., 1993; Farhanah et al., 2018; Smith et al., 2015b), MDV (Burgess et al., 2001; Cole, 1968; Mohd Isa et al., 2020) and AIV (Ruiz‐Hernandez et al., 2016) infections were referenced.

### Identification of chicken interferon genes of interest

Ensembl genome browser release 103 (https://www.ensembl.org/index.html) was used to identify the interferon pathway genes of interest and their genomic co‐ordinates. Genomic positions from this release were used for interrogating sequence data from the research lines mapped to the GRCg6a chicken reference genome (GenBank assembly accession number GCA_000002315.5). Paralogues with >95% target identity were also identified and examined in this study (Gheyas et al., 2015).

### Whole genome sequence data from the research lines

Genomic data from the research lines is the same as that used in the development of the 600 K chicken SNP genotyping array (Kranis et al., 2013). Briefly, 10 chickens from each line were supplied by the Institute of Animal Health (Compton, UK; now available through the National Avian Research Facility (NARF, Edinburgh, UK), with all chickens within lines being of the same gender (female in all lines except for male line N chickens). DNA was extracted from blood samples and pooled for sequencing on an Illumina GAIIx platform using a paired‐end protocol where read length was 101 bases. These previously generated whole genome sequence data from each line were aligned to the GRCg6a chicken reference genome using the Burrows‐Wheeler Aligner mem (BWA‐mem) software package, version 0.7.15, using default settings (Li & Durbin, 2009).

### Variant calling and annotation

The genome analysis toolkit (gatk) version 3.8 and Picard 2.9.2 packages were used throughout the variant calling process following the best practices workflow for germline short variant discovery (gatk, n.d.). Picard’s SortSam function was used to sort the mapping data into coordinate order and the MarkDuplicates function highlighted duplicate reads. Base Quality Score Recalibration of the mapped reads was then performed using the gatk. Over 20 million known single nucleotide polymorphisms (SNPs) in the chicken genome from Ensembl (release 92) were used in the BaseRecalibrator step as known variants to be masked from consideration as sequencing errors during the recalibration process. gatk’s HaplotypeCaller function was used to call short variants. This resulted in a GVCF file for each line, and the files were then combined into one VCF file using the GenotypeGVCF function. The variant quality score recalibration (VQSR) approach was applied for filtering variants (gatk, n.d.). For this, over 1 million validated polymorphic SNPs (Kranis et al., 2013) were used as a truth dataset alongside the previous known dataset and the unfiltered variants from the research lines to create a trained Gaussian mixture model using VariantRecalibrator. The model calculated a variant quality score log‐odds (VQSLOD) score for each SNP in the truth dataset and the research lines dataset. The ApplyRecalibration step of VQSR (also called ‘ApplyVQSR’ in the latest version of the gatk) was used with a sensitivity filter of 99% to remove SNPs with a VQSLOD score lower than the top 99% of the truth set, as these are likely to be false positives. To further minimise the inclusion of false positives, SNPs with a VQSLOD score <20 were removed.

The ensembl variant effect predictor (VEP) webtool (https://www.ensembl.org/info/docs/tools/vep/index.html) was used to annotate SNPs identified within and around the genes of interest. Variants were defined as upstream (if within 1 kb upstream of a transcription start site), downstream (within 1 kb downstream of the gene), 3′ untranslated region (3′UTR), 5′ untranslated region (5′UTR), intronic, synonymous or missense variants (McLaren et al., 2016).

### Prediction of potential functional impacts of SNPs

To determine the potential impact of missense variants on protein function and amino acid sequences, corresponding variants were examined using the normal‐smart, sift‐sequence, provean protein and snap2 webtools. normal‐smart (Simple Modular Architecture Research Tool; http://smart.embl‐heidelberg.de) predicts domains within the protein sequence. Variants affecting particular protein domains could therefore be identified (Letunic et al., 2021). sift‐sequence uses the sift (Sorting Intolerant From Tolerant) algorithm to predict whether an amino acid substitution, owing to a missense SNP, affects protein function based on sequence evolutionary conservation and the physical properties of each amino acid. Default settings were used for the prediction of the sift score for each missense SNP (Sim et al., 2012). Similarly, provean protein (Protein Variation Effect Analyser; http://provean.jcvi.org/seq_submit.php) is another method to investigate the impact of amino acid substitution on the biological function of a protein (Choi & Chan, 2015) and again the predictions were made using default settings. snap2 (Screening for Non‐Acceptable Polymorphisms; https://rostlab.org/services/snap2web/) uses a neural network‐based classifier for distinguishing between functional and neutral variants within the protein sequence where the prediction algorithm is trained using more than 100 000 experimentally annotated variants from multiple online databases (PMD, SWISS‐PROT, OMIM) and HumVar (Hecht et al., 2015).

To determine the potential impact of variants on micro‐RNA (miRNA) binding sites, 3′UTR variants were compared with miRNA binding sites identified via the miRDB (http://www.mirdb.org) online database (Chen & Wang, 2020). The potential impact of variants on transcription factor binding sites (TFBSs) was investigated by uploading 1 kb of upstream gene sequences into the MATCH 1.0 public webserver (http://gene‐regulation.com/pub/programs.html#match). SNP positions were compared with known TFBSs and any changes recorded. Parameters used were ‘high quality matrices only’ and to ‘minimise false positives’ (Kel et al., 2001).

To determine the impact of variants on the mature mRNA secondary structure, SNPs identified as 3′UTR, missense, synonymous and 5′UTR were analysed using the rnasnp (https://rth.dk/resources/rnasnp/) and snpfold (http://ribosnitch‐ compute.bio.unc.edu/snpfold/SNPfold.html) webtools alongside the mRNA sequence of the affected gene of interest. rnasnp assesses the effect of an SNP on a RNA secondary structure, as structural characteristics are essential for the correct functioning of RNA molecules (Sabarinathan et al., 2013). The rnasnp predictions were made using the ‘Mode 2’ function, which uses a local folding algorithm designed for long RNA molecules. snpfold considers all possible RNA conformations for the input sequence and uses Pearson correlation coefficients to compare secondary structure change between wild‐type and variant sequences (Halvorsen et al., 2010). The *p*‐values indicating significance generated by these software tools were corrected for multiple tests using Bonferroni correction.

## RESULTS

### Phenotype definition of chicken lines

Previous observations of resistant and susceptible phenotypes in response to strains of IBV, IBDV, MDV and AIV for the research lines investigated in this study are described in Table 1. Resistance and susceptibility were determined based on variables such as viral titre, mortality, clinical symptoms and transmissibility. Issues encountered when considering such studies included the subjective nature of how resistance and susceptibility are defined and the lack of systematic study of these lines against the different viruses. These observations have also been made at different time points of infection, different chicken ages and while using different virus strains and infection doses or routes, adding further complexity to an already complex trait. The different MHC backgrounds of each line also need to be considered (Stone, 1975). The phenotypes defined were used to consider SNPs identified in the context of resistance and susceptibility depending on how they are distributed between lines.

**TABLE 1:** Resistant and susceptible phenotypes of research lines

Inbred chicken line	MHC B haplotype	Virus resistance phenotype (Resistant/Moderate susceptibility/Susceptible)			
		IBV (Bumstead et al., 1989; Cook et al., 1990; Nakamura et al., 1991; Otsuki et al., 1990)	IBDV (Asfor et al., 2021; Bumstead et al., 1993; Farhanah et al., 2018; Smith et al., 2015b)	MDV (Burgess et al., 2001; Cole, 1968; Mohd Isa et al., 2020)	LPAIV (Ruiz‐Hernandez et al., 2016)
Wl	B14	M	S	–	–
15I	B15	S	R	S	–
7_2_	B2	S	R	S	–
6_1_	B2	M	M	R	–
C	B4 and B12	R	R	–	S
N	B21	R	M	R	–
0	B21	–	R	–	R
P2a	B19	–	–	S	–

LPAIV ‐ Lowly Pathogenic Avian Influenza Virus; “R” denotes resistance, “M” denotes moderate susceptibility, “S” denotes susceptibility and “–” indicates no data identified.

These data do not account for potential gender differences.

These lines were originally housed at the Institute for Animal Health, Compton, UK, but are now available at the NARF, Roslin, UK.

### Chicken interferon pathway genes

The genes of interest, the paralogues of these genes and their location in the GRCg6a reference genome are shown in Table 2. As the JAK–STAT pathway is intrinsic to ISG regulation, the *TYK2*, *JAK1*, *JAK2*, *STAT1*, *STAT2* and *IRF9* genes were examined along with the interferons and their receptors. Seventeen genes of interest were identified. *IFNα* and *IFNλ* were found to have 11 and three paralogues respectively, resulting in 29 genomic locations being explored. It should be noted that *IFNα*, *IFNβ*, *IFNκ* and *JAK2* are all located on the Z sex chromosome. In birds the female is the heterogametic sex, having ZW sex chromosomes, whereas the male has two copies of the Z chromosome. Incomplete dosage compensation in chickens results in higher expression of Z chromosome genes in males (Garcia‐Morales et al., 2015).

**TABLE 2:** Genes of interest identified in GRCg6a

Gene	Gene/paralogue ID	Sequence location and direction
*IFNλ*	ENSGALG00000052146	Chromosome 7: 4610682‐4 612 191 forward strand
	ENSGALG00000050947	Chromosome 7: 4572389‐4 573 897 forward strand
	ENSGALG00000047344	Chromosome 7: 4591535‐4 593 042 forward strand
*IFNα*	ENSGALG00000048874	Chromosome Z: 7395233‐7 395 995 forward strand
	ENSGALG00000052209	Chromosome Z: 7410282‐7 411 044 forward strand
	ENSGALG00000044725	Chromosome Z: 7399231‐7 399 942 forward strand
	ENSGALG00000047630	Chromosome Z: 7381503‐7 382 265 forward strand
	E*NSGALG00000050924*	Chromosome Z: 7401271‐7 402 033 forward strand
	ENSGALG00000053752	Chromosome Z: 7387284‐7 388 046 forward strand
	ENSGALG00000054396	Chromosome Z: 7385475‐7 386 237 forward strand
	ENSGALG00000046996	Chromosome Z: 7377531‐7 378 293 forward strand
	ENSGALG00000053207	Chromosome Z: 7414251‐7 415 013 forward strand
	ENSGALG00000054368	Chromosome Z: 7391261‐7 392 023 forward strand
	ENSGALG00000054104	Chromosome Z: 7421956‐7 422 718 forward strand
*IFNβ*	ENSGALG00000005759	Chromosome Z: 7372029‐7 372 640 forward strand
*IFNκ*	ENSGALG00000015062	Chromosome Z: 34282011‐34 285 224 reverse strand
*IFNγ*	ENSGALG00000009903	Chromosome 1: 35173604‐35 177 772 reverse strand
*IFNAR1*	ENSGALG00000030363	Chromosome 1: 106613562‐106 632 348 forward strand
*IFNAR2*	ENSGALG00000041867	Chromosome 1: 106580169‐106 592 227 forward strand
*IFNLR1*	ENSGALG00000004231	Chromosome 23: 5819774–5 826 643 reverse strand
*IL‐10Rβ*	ENSGALG00000037989	Chromosome 1: 106593970‐106 604 799 forward strand
*IFNGR1*	ENSGALG00000013865	Chromosome 3: 54895190‐54 914 161 forward strand
*IFNGR2*	ENSGALG00000032660	Chromosome 1: 106645075‐106 653 536 forward strand
*TYK2*	ENSGALG00000030599	Chromosome 30: 178603–197 548 forward strand
*JAK1*	ENSGALG00000011031	Chromosome 8: 28552333‐28 603 383 reverse strand
*JAK2*	ENSGALG00000015027	Chromosome Z: 27532087‐27 615 380 forward strand
*STAT1*	ENSGALG00000007651	Chromosome 7: 7912708‐7 932 691 reverse strand
*STAT2*	ENSGALG00000030661	Chromosome 33: 6934615‐6 940 207 forward strand
*IRF9*	ENSGALG00000030291	Chromosome 20: 10063682‐10 066 572 reverse strand

### SNPs across research lines identified in GRCg6a

Prior to VQSR, 12142027 SNPs were identified between the GRCg6a reference genome and the research lines. Over 81% of the SNPs (9862336) passed VQSR and 79% (9584464) had a VQSLOD score >20. Of these, 1760 were found to occur within the defined locations of the genes of interest, including 1 kb up‐ and downstream of the genes. The distribution of these SNPs after being defined by VEP can be seen in Table 3. The SNPs are split into the following annotation categories: 81.4% (1432) intronic, 5.74% (101) upstream, 5.17% (91) downstream, 2.95% (52) 3′UTR, 2.33% (41) synonymous, 2.05% (36) missense and 0.40% (7) 5′UTR SNPs. Of the 29 genomic locations examined (Table 2), only 16 were found to contain one or more SNPs when research lines were compared with the GRCg6a reference.

**TABLE 3:** Distribution of SNPs in genes of interest across lines

Distribution of SNPs in genes of interest across lines			Upstream	Downstream	5′UTR	3′UTR	Intronic SNPs	Synonymous SNPs	Missense SNPs
Gene affected	Transcript ID	Total SNPs in gene (±1 kb)							
*IFNγ*	*ENSGALT00000016105.4*	53	6	13	0	2	32	0	0
*IFNα*	*ENSGALT00000102661.1*	8^a^	8	0	0	0	0	0	0
*IFNβ*	*ENSGALT00000039477.2*	10^a^	3	5	0	0	0	1	1
*IFNκ*	*ENSGALT00000032834.4*	9^a^	1	1	0	2	4	1	0
*IFNAR1*	*ENSGALT00000064880.2*	208	9	13	1	15	160	4	6
*IFNAR2*	*ENSGALT00000046822.2*	185	20	16	1	6	133	1	8
*IFNLR1*	*ENSGALT00000098112.1*	16	1	1	2	1	7	1	3
*IL‐10Rβ*	*ENSGALT00000073701.2*	169	18	12	0	7	126	2	4
*IFNGR1*	*ENSGALT00000031776.5*	163	9	8	1	7	136	0	2
*IFNGR2*	*ENSGALT00000061931.2*	85	5	13	0	11	53	2	1
*TYK2*	*ENSGALT00000058693.2*	23	0	1	0	0	15	1	6
*JAK1*	*ENSGALT00000017969.6*	626	11	5	2	1	591	14	2
*JAK2*	*ENSGALT00000024235.6*	143	2	1	0	0	136	4	0
*STAT1*	*ENSGALT00000057169.2*	21	3	0	0	0	17	0	1
*STAT2*	*ENSGALT00000050242.2*	12	0	2	0	0	7	3	0
*IRF9*	*ENSGALT00000100907.1*	29	5	0	0	0	15	7	2
	Total SNPs	1760	101	91	7	52	1432	41	36
		SNP % distribution	5.74	5.17	0.398	2.95	81.4	2.33	2.05

^a^ The low number of identified SNPs may be a reflection of the low sequence coverage in these regions.

Only one of the *IFNα* paralogues contained SNPs, all of which were upstream of the gene. *IFNλ* and its two paralogues were also not found to contain SNPs. It was considered that this may be due to these genes being highly conserved. However, this finding prompted examination of the sequence read depth of these genes in each line, and this identified low coverage for these regions. For example, the *IFNλ* paralogue (ENSGALG00000050947) with the highest coverage across all lines had an average read depth of only 1.55x. Compared with a longer gene with no paralogues, such as *IFNAR1*, the average read depth across lines was found to be 15×. The issues of coverage observed are probably due to the presence of multiple similar paralogues in the genome and the short length of reads (101 bp) used in this study, as this will result in failure to distinguish unique genomic regions during read alignment. High GC content in particular regions will also mean problems with sequence coverage. Given this finding and the importance of both IFNα and IFNλ in these pathways it would be advisable to carry out long‐read sequencing where reads can cover the entire gene region, as this would allow unique reads to be mapped and variants to be identified.

However, many more SNPs were detected in our current analysis using GRCg6a compared with the previous analysis that used the Galgal4 reference genome (Kranis et al., 2013). The comparison is shown in Appendix S2. The difference in the number reflects the better assembly and more complete build of GRCg6a, compared with Galgal4. For example, in Galgal4 no SNPs were detected from genes on chr30 or 33 but in GRCg6a we detected many SNPs (e.g. from genes TYK2 and STAT2). Moreover, in our current analysis we have applied a more advanced variant calling approach (using gatk’s VQSR method for filtration) compared with the hard filtration applied in the previous analysis.

### Prediction of functional impacts of missense SNPs identified from genes of interest

**FIGURE 2:**
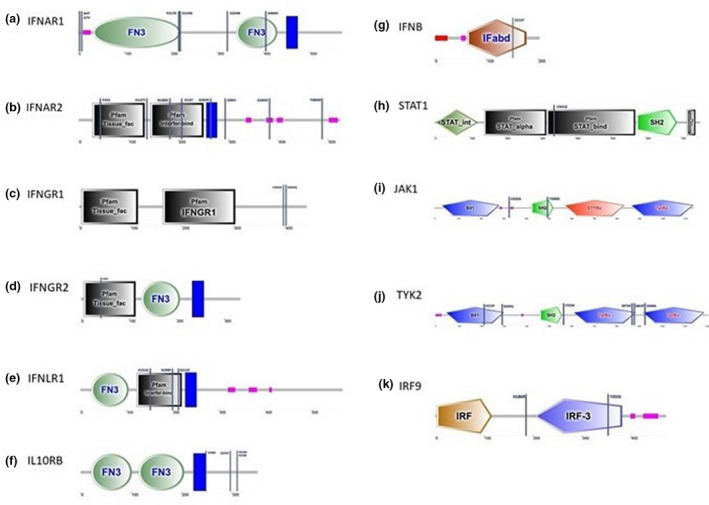
Location of missense SNPs with regards to predicted protein domains. (a) IFNAR1 – Y217, E219G and A404V can be found in fibronectin type 3 (FN3) domains. (b) IFNAR2 – P45S occurs in a tissue factor domain, N188D and I214T occur in an interferon binding domain and G264V occurs in the transmembrane region. (c) IFNGR1 – no SNPs were identified in the predicted domains. (d) IFNGR2 – C44R in tissue factor domain. (e) IFNLR1 – E151G, S199P and S211P occur in an interferon binding domain. (f) IL10Rβ – no SNPs were identified in predicted domains. (g) IFNβ – S151P SNP occurs in an IFNα/β/δ domain. (h) STAT1 – V341G SNP occurs in a STAT binding domain. (i) JAK1 – Y500D occurs in an Src Homolog 2 (SH2) domain. (j) TYK2 – H215P and R299Q occur in a band 4.1 (B41) domain and S875N, S883P and D908A occur in tyrosine kinase catalytic domains (TyrKc). (k) IRF9 – Y335S occurs in an interferon regulatory factor 3 (IRF3) domain. Note, chickens lack IRF3 but have an IRF7 orthologue. IF*αβδ*, Interferon alpha, beta and delta domain; STAT_int, STAT protein interaction domain; STAT_alpha, STAT protein all‐alpha domain; STAT_bind, STAT protein DNA binding domain; B41, band 4.1 homologue domain (also known as ERM domain, erzin/radixin/moesin domain); STYKc, protein kinase of unclassified specificity; IRF, interferon regulatory factor domain. Pink indicates low complexity regions and dark blue blocks signify transmembrane regions

The potential functional consequences of identified missense SNPs (*n* = 36) as predicted by sift, provean and snap2 scores can be found in Table 4. Those predicted to have a deleterious effect are shown in bold. A sift or provean score of <0.05 or − 2.5 respectively, indicates a predicted deleterious change, whereas a snap2 score of greater than zero predicts an impact on protein function. Thirteen of the amino acid substitutions were predicted to have an effect by at least one of the webtools, with five of these being predicted by two and the Y500D substitution in *JAK1* predicted to have an effect by all three. The locations of all 36 SNPs predicted protein domains impacted can be seen in Figures 2a–k.

**TABLE 4:** Missense SNPs in genes of interest identified in chicken lines

SNP genome coordinate	Protein affected	Line(s) in which SNP is present	Alleles (ref/SNP)	Amino acid substitution	SIFT score	PROVEAN score	SNAP2 score	Comments on SNP location
Chr1:106,613,646	IFNAR1	6_1_, 7_2_, 0	G/A	A4T	0^b^	0.012	−84	
Chr1:106,613,656	IFNAR1	7_2_	C/T	A7V	0^b^	0.647	−25	
Chr1:106,621,892	IFNAR1	15I, 6_1_, 7_2_, C, N, P2a^a^, 0, Wl	T/G	Y217D	0.29	0.363	**32**	Occurs in FN3 domain
Chr1:106,621,899	IFNAR1	N	A/G	E219G	0.44	−0.719	**18**	Occurs in FN3 domain
Chr1:106,623,894	IFNAR1	15I, 6_1_, 7_2_, C, N, 0, Wl	G/A	S324N	1	1.099	−81	Occurs in tissue factor domain
Chr1:106,625,990	IFNAR1	0^a^	C/T	A404V	0.76	0.729	−87	Occurs in interferon binding domain
Chr1:106,587,598	IFNAR2	C	C/T	P45S	0^b^	0.759	−78	Occurs in tissue factor domain
Chr1:106,588,380	IFNAR2	15I, 7_2_, C, 0^a^, Wl	C/T	H137Y	1	0.037	−65	
Chr1:106,588,700	IFNAR2	15I, 7_2_, P2a^a^, 0^a^, Wl	A/G	N188D	0.7	−0.238	−89	Occurs in interferon binding domain
Chr1:106,589,401	IFNAR2	15I, P2a^a^, 0, Wl	T/C	I214T	0.11	−0.435	−68	Occurs in interferon binding domain
Chr1:106,589,970	IFNAR2	15I, C, P2a^a^, 0^a^, Wl	G/T	G264V	0.15	0.584	−1	Occurs in transmembrane region
Chr1:106,590,778	IFNAR2	15I, C, P2a^a^, 0, Wl	A/G	I296V	1	−0.005	−75	
Chr1:106,591,040	IFNAR2	15I, C, P2a^a^, 0^a^, Wl	G/A	G383D	0.69	1.243	−29	
Chr1:106,591,349	IFNAR2	15I, C, P2a^a^, 0, Wl	C/T	T486M	^aa^0	−0.991	−62	
Chr3:54,913,306	IFNGR1	15I, 6_1_, 7_2_, C, 0, Wl	T/C	V395A	0.27	−0.16	−16	
Chr3:54,913,327	IFNGR1	15I, 6_1_, 7_2_, C, 0, Wl	G/A	R402Q	0.48	1.272	−71	
Chr1:106,647,185	IFNGR2	6_1_	T/C	C44R	0.62	3.391	−60	Occurs in tissue factor domain
Chr23:5,823,279	IFNLR1	N^a^	T/C	E151G	0^b^	−1.341	−19	Occurs in interferon binding domain
Chr23:5,822,624	IFNLR1	C^a^	A/G	S199P	0.23	−1.046	**14**	Occurs in interferon binding domain
Chr23:5,822,588	IFNLR1	7_2_ ^a^, C^a^, 0^a^	A/G	S211P	0.06	−**4.218**	**41**	Occurs in interferon binding domain
Chr1:106,602,130	IL10RB	6_1_	T/G	S248A	1	0.375	−66	
Chr1:106,603,837	IL10RB	6_1_, 7_2_, N, P2a^a^, 0^a^	A/T	Q293H	**0.04**	−1.17	−95	
Chr1:106,603,910	IL10RB	7_2_	A/G	R318G	0.38	−0.716	**9**	
Chr1:106,603,911	IL10RB	C, P2a^a^	G/A	R318K	1	−0.092	−78	
ChrZ:7,372,479	IFNβ	N^a^	T/C	S151P	0.36	−1.728	**52**	Occurs in IFNα/β/δ domain
Chr7:7,923,855	STAT1	15I^a^, 7_2_ ^a^, Wl^a^	A/C	V341G	0.03^b^	−**4.766**	**39**	Occurs in STAT binding domain
Chr8:28,566,979	JAK1	N^a^, P2a^a^, 0	A/G	V332A	0.67	−0.305	−67	
Chr8:28,564,446	JAK1	C^a^, Wl^a^	A/C	Y500D	**0.04**	−**3.757**	**61**	Occurs in SH2 domain
Chr30:182,787	TYK2	7_2_ ^a^, Wl^a^	A/C	H215P	0.2	−**2.732**	**63**	Occurs in B41 domain
Chr30:183,686	TYK2	15I^a^, 6_1_, 7_2_, N, P2a, 0, Wl	G/A	R299Q	0.44	−0.551	−17	Occurs in B41 domain
Chr30:186,858	TYK2	7_2_ ^a^, C^a^, 0^a^	T/C	V550A	**0.01**	−**2.65**	−26	Occurs in SH2 domain
Chr30:190,505	TYK2	6_1_, 7_2_, P2a, 0^a^	G/A	S875N	0.45	0.351	−96	Occurs in TyrKc domain
Chr30:190,528	TYK2	C^a^	T/C	S883P	0.25	−**3.994**	**12**	Occurs in TyrKc domain
Chr30:190,801	TYK2	6_1_ ^a^	A/C	D908A	0.34	−1.029	−53	
Chr20:10,064,956	IRF9	P2a^a^	T/C	H186R	0.51	−1.24	−38	
Chr20:10,064,260	IRF9	6_1_ ^a^, C^a^, 0^a^	T/G	Y355S	0.15	−**4.102**	**63**	Occurs in IRF‐3 domain

Scores in bold indicate a predicted significant effect.

^a^ SNP occurs in only one allele and is therefore heterozygous.

^b^ SIFT scores with low confidence in the value due to too few comparable sequences for the software to make a reliable prediction.

Considering the different research lines, there are 42 instances where it could be inferred that a missense SNP contributes to resistance to one of the viruses, whereas 47 instances could be related to susceptibility. The arguments for these can be found in Table S3 (see the worksheet missenseSNPs). For example, the aforementioned Y500D substitution, visible in Figure 2i, occurs in lines C and W1 and affects the normal‐smart‐predicted SH2 domain of JAK1. It could be hypothesised that this SNP contributes to IBV resistance or AIV susceptibility in line C, whereas in Wl it could be linked to IBDV susceptibility. Of the 36 SNPs, 13 were found to be heterozygous. The remaining 23 SNPs were homozygous in at least one line. This finding could have further implications as allele‐specific expression could potentially affect wild‐type vs. mutant protein function.

### SNPs potentially affecting mRNA secondary structure

Twenty‐one of the 136 SNPs occurring in the mRNA sequence among the lines were predicted to affect mRNA secondary structure by at least one of the webtools (rnasnp and/or snpfold with *p* < 0.1; Table 5). Only one SNP in the 3′UTR region of *IFNκ* (ChrZ:34282637) was indicated to significantly impact secondary structure by both webtools. This SNP occurs in the AIV resistant line 0, but is absent from the susceptible C line; therefore it could be perceived that this variation affects the *IFNκ* mRNA in a way that may contribute to AIV resistance. In the context of viral infection and how the SNPs distribute between the lines considered in this study, 31 instances of these could be related to viral resistance, whereas 21 instances could be related to susceptibility (Table S3; worksheet mRNAstructureAlteringSNPs).

**TABLE 5:** SNPs of interest predicted to potentially perturb mRNA secondary structure

SNP genome coordinate	mRNA affected	Line(s) in which SNP is present	Alleles (ref/SNP)	mRNA base change	RNAsnp P‐value	SNPfold P‐value	Region affected
Chr1:106613656	*IFNAR1*	7_2_	C/T	C/U	0.4067	**0.0078**	Exonic
Chr1:106622867	*IFNAR1*	N, 0^a^	C/T	C/U	0.6077	**#0.016**	Exonic
Chr1:106623894	*IFNAR1*	15I, 6_1_, 7_2_, C, N, 0, Wl	G/A	G/A	**0.0446**	0.4078	Exonic
Chr1:106625990	*IFNAR1*	0^a^	C/T	C/U	0.5257	**0.0808**	Exonic
Chr1:106631527	*IFNAR1*	P2a	G/A	G/A	0.8683	**0.0086**	3′UTR
Chr1:106632281	*IFNAR1*	15I, 6_1_, 7_2_, C, N, P2a^a^, 0 WL	T/C	U/C	0.9474	**0.0093**	3′UTR
Chr1:106589970	*IFNAR2*	15I, C, P2a^a^, 0^a^, Wl	G/T	G/U	0.424	**0.046**	Exonic
Chr1:106592036	*IFNAR2*	15I, C, 0, Wl	T/G	U/G	0.5046	**0.0275**	3′UTR
Chr3:54913306	*IFNGR1*	15I, 6_1_, 7_2_, C, 0, Wl	T/C	U/C	**0.084**	0.3036	Exonic
Chr1:106652516	*IFNGR2*	N, 0^a^	G/A	G/A	0.9103	**0.0198**	3′UTR
Chr1:106653379	*IFNGR2*	N, 0^a^	C/T	C/U	0.1912	**0.0473**	3′UTR
Chr23:5823279	*IFNLR1*	N^a^	T/C	A/G	**0.0811**	0.3326	Exonic
Chr1:106604195	*IL10RB*	7_2_ ^a^, C^a^	T/G	U/G	**0.045**	0.2181	3′UTR
Chr1:106604577	*IL10RB*	15I, 0^a^, Wl	G/A	G/A	**0.0565**	0.3225	3′UTR
ChrZ:34282637	*IFNK*	6_1_, 7_2_, N, P2a, 0, Wl	C/T	G/A	**#0.027**	**0.0968**	3′UTR
Chr8:28553812	*JAK1*	15I, 7_2_, C, N^a^, P2a^a^	G/A	C/U	**0.0978**	0.591	Exonic
Chr8:28559109	*JAK1*	C	A/G	U/C	**0.056**	0.4255	Exonic
Chr8:28564450	*JAK1*	15I, 6_1_, 7_2_, C, N^a^, P2a^a^, Wl	T/G	A/C	**0.0972**	0.4388	Exonic
Chr8:28566979	*JAK1*	N^a^, P2a^a^, 0	A/G	U/C	0.1547	**0.0105**	Exonic
Chr8:28567177	*JAK1*	P2a^a^	T/C	A/G	0.7025	**0.0056**	Exonic
Chr8:28576480	*JAK1*	N^a^, P2a^a^, 0	C/T	G/A	**0.051**	0.3395	Exonic

Allele changes are listed for the forward strand; *IFNLR1*, *IFNκ* and *JAK1* are found on the reverse strand so the mRNA base changes observed in these genes reflect that of the template strand.

Scores in bold can be considered significant (*p* > 0.1), # – scores still significant after Bonferroni correction.

^a^ SNP occurs in only one allele and is therefore heterozygous.

### SNPs in micro‐RNA and transcription factor binding sites potentially contribute to resistance and susceptibility phenotypes

Three SNPs were found to impact either a TF or a miRNA binding site, as listed in Table 6. The gga‐miR‐1627‐3p and gga‐miR‐196‐1‐3p binding sites in the 3′UTR of *IFNAR1* are seen to be affected, whereas a V‐MAF binding site upstream of *STAT1* is also disrupted. The miRNA gga‐miR‐196‐1‐3p is believed to have multiple roles including embryonic development and feather follicle development (Chen et al., 2017; Xu et al., 2013), whereas the V‐MAF TF is a viral oncogene (Nishizawa et al., 1989). In the context of viral infection and how the SNPs are distributed between the lines, there are five instances where viral resistance can be inferred, and two instances could be linked to susceptibility (Table S3, worksheet: TF‐miRNA‐bindingSNPs). For example, the SNP upstream of *STAT1* potentially affecting V‐MAF binding occurs in the MDV resistant line 6_1_ but is absent from the susceptible lines 7_2_ and 15I.

**TABLE 6:** SNPs of interest predicted to affect TF or miRNA binding sites

SNP genome coordinate	Gene/mRNA affected	Line(s) in which SNP is present	Alleles (ref/SNP)	mRNA base change	Comments
Chr1:106631586	*IFNAR1*	0 ^a^	C/T	C/U	Identified as gga‐miR‐1627‐3p binding site
Chr1:106632281	*IFNAR1*	15I, 6_1_, 7_2_, C, N, P2a^a^, 0, Wl	T/C	U/C	Identified as gga‐miR‐196‐1‐3p binding site
Chr7:7933106	*STAT1*	6_1_, C, P2a^a^, 0^a^	C/T	N/A	Identified as V‐MAF TF binding site

^a^ SNP occurs in only one allele and is therefore heterozygous.

## DISCUSSION

Considering that the interferon pathways play a key role in immune responses, not only to viral infections, but also bacterial and parasitic infections, it is plausible that variations in the genes involved may contribute to differences in resistance and susceptibility. Here, we analysed genome sequence data from eight chicken research lines and identified variants from interferon pathway genes. The impact of these SNPs was considered with regard to their potential functional effects and distribution amongst the lines. This process resulted in a total of 51 potentially relevant SNPs being identified. When considered in terms of the impact of the SNP and in the context of specific virus response, 78 instances could be related to resistance and 70 instances could be related to susceptibility. Table 7 highlights 19 SNPs of interest that correlate with resistance or susceptibility to at least two of the viruses within the scope of this study. Furthermore, prediction tools identified five of these 19 SNPs as having a significant impact on protein function or mRNA folding.

**TABLE 7:** SNPs of interest correlating with resistance or susceptibility against more than one virus

SNP genome coordinate	Gene/mRNA affected	Phenotype (resistant/susceptible)	Virus affected (line with phenotype)	Comments
Chr1:106603837	*IL10RB*	Resistant	AIV (0) IBDV (7_2_, 15L) MDV (N, 6_1_)	Significant SIFT score of 0.04 Heterozygous in line 0
Chr1:106621899	*IFNAR1*	Resistant	IBV (N) MDV (N)	Significant SNAP2 score of 18
Chr1:106622867	*IFNAR1*	Resistant	AIV (0) IBDV (0) IBV (N) MDV (N)	SNPfold P‐value = 0.016 – significant after Bonferroni correction for *p* < 0.1 Heterozygous in line 0
Chr1:106625990	*IFNAR1*	Resistant	AIV (0) IBDV (0)	SNPfold P‐value = 0.0808 – not significant after applying Bonferroni correction Heterozygous in line 0
Chr1:106652516	*IFNGR2*	Resistant	AIV (0) IBDV (0) IBV (N) MDV (N)	SNPfold P‐value = 0.0198 – not significant after applying Bonferroni correction Heterozygous in line 0
Chr1:106653379	*IFNGR2*	Resistant	AIV (0) IBDV (0) IBV (N) MDV (N)	SNPfold P‐value = 0.0473 – not significant after applying Bonferroni correction Heterozygous in line 0
Chr23:5823279	*IFNLR1*	Resistant	IBV (N) MDV (N)	SNPfold P‐value = 0.0811 – not significant after applying Bonferroni correction Heterozygous in line N
Chr8:28566979	*JAK1*	Resistant	IBDV (0) AIV (0) IBV (N)	SNPfold P‐value = 0.0105 – not significant after applying Bonferroni correction Heterozygous in line N
Chr8:28576480	*JAK1*	Resistant	AIV (0) IBDV (0) IBV (N)	RNAsnp P‐value = 0.051 – not significant after applying Bonferroni correction Heterozygous in line N
ChrZ:7372479	*IFNβ*	Resistant	IBV (N) MDV (N)	Significant SNAP2 score of 52 Heterozygous in line N
Chr1:106588380	*IFNAR2*	Susceptible	MDV (15L, 7_2_) AIV (C)	No impact calculated by functional prediction tools
Chr1:106589970	*IFNAR2*	Susceptible	MDV (15L, P2a) AIV (C) IBDV (Wl)	SNPfold *p*‐value = 0.046 – not significant after applying Bonferroni correction Heterozygous in line P2a
Chr1:106590778	*IFNAR2*	Susceptible	MDV (15L, P2a) IBDV (Wl)	No impact calculated by functional prediction tools Heterozygous in line P2a
Chr1:106591040	*IFNAR2*	Susceptible	MDV (15L, P2a) AIV (C) IBDV (Wl)	No impact calculated by functional prediction tools Heterozygous in line P2a
Chr1:106591349	*IFNAR2*	Susceptible	MDV (15L, P2a) IBDV (Wl)	No impact calculated by functional prediction tools Heterozygous in line P2a
Chr1:106592036	*IFNAR2*	Susceptible	MDV (15L) IBDV (Wl)	SNPfold P‐value = 0.0275 – not significant after applying Bonferroni correction
Chr30:182787	*TYK2*	Susceptible	IBV (7_2_) MDV (7_2_) IBDV (Wl)	Significant PROVEAN score of −2.732 and significant SNAP2 score of 63 Heterozygous in lines 7_2_ and Wl
Chr7:7923855	*STAT1*	Susceptible	IBV (15L, 7_2_) MDV (15L, 7_2_) IBDV (Wl)	Significant PROVEAN score of −4.766 and significant SNAP2 score of 39 Heterozygous in lines 15L, 7_2_ and Wl
Chr8:28564450	*JAK1*	Susceptible	AIV (C) IBDV (Wl)	RNAsnp P‐value = 0.0972 – not significant after applying Bonferroni correction

It is possible to hypothesise the mechanism by which some of these SNPs may act given their effect. Missense SNPs result in an amino acid change in the polypeptide sequence and this may have consequences on the final protein structure, possibly affecting features such as substrate binding efficiency, protein–protein complex interfaces or catalytic activity. The SNP at position Chr30:182787 where histidine is replaced by proline (H215P) lies in a predicted band 4.1 (B41) domain of TYK2. This SNP is present in the IBV‐ and MDV‐susceptible line 7_2_ and the IBDV‐susceptible line Wl and may be a contributing factor to these phenotypes. For example, the change from the positively charged, polar histidine to the neutral, non‐polar proline may result in a conformational change that negatively impacts the process of phosphorylation of the STAT proteins, resulting in reduced overall upregulation of ISGs. This change could potentially explain why line 7_2_ developed more progressive MD compared with lines 15 L, 6_2_ and N (Burgess et al., 2001), or why greater mortality was seen in line Wl chickens infected with IBDV compared with various other lines (Bumstead et al., 1993).

The effect of a SNP on protein function can be executed not just by alteration of the amino acid but also by affecting the mRNA secondary structure. Exonic (both synonymous and missense), 5′UTR and 3′UTR SNPs may affect a transcript’s secondary structure by altering base pairing within the mRNA. Such structural alteration may affect mRNA stability. Altered secondary structure around the 5′UTR region may impact translation initiation efficiency by affecting the ability of the ribosome to bind to the mRNA, whereas secondary structure changes in the 3′UTR region may affect the accessibility of miRNAs to binding sites, impacting their ability to silence translation in the mRNA (Haas et al., 2012; Mauger et al., 2019). In this study, an SNP at position Chr1:106652516 was identified that results in a guanine to adenine change in the 3′UTR region of the *IFNGR2* mRNA in the IBDV‐resistant line N and was predicted to significantly affect the secondary structure. *IFNGR2* exhibits greater levels of expression after infection with virulent IBDV (vvIBDV; Farhanah et al., 2018), which would indicate the importance of this gene and the type II interferon pathway. It is possible that this SNP in line N may synergise with this upregulation by preventing miRNA binding owing to an altered secondary structure, leading to greater levels of translation and a greater type II pathway response compared with lines lacking this SNP, where miRNA binding and silencing may still occur.

SNPs can also directly affect miRNA binding sites in the 3′UTR of the mRNA by altering the recognition sequence, potentially impacting miRNA binding of the transcript and the resultant silencing effect of this interaction. Using miRDB, it was seen that potential miRNA binding sites for gga‐miR‐1627‐3p and gga‐miR‐196‐1‐3p in the 3′UTR of *IFNAR1* were altered by SNPs at positions Chr1:106632281 and Chr1:106631586 respectively. *Gga‐miR‐1627‐3p* was previously found to exhibit greater expression in macrophages with the MHC haplotype B19 compared with those with B2, suggesting that it may play a role in immune response (Irizarry et al., 2017). The SNP affecting the gga‐miR‐1627‐3p binding site only occurs in line 0, which is perceived as resistant to AIV, so it is therefore plausible that this SNP could contribute to this resistant phenotype. Interferons were discovered owing to their ability to inhibit influenza virus (Isaacs & Lindenmann, 1957) and studies using type I interferons as both a pre‐treatment and treatment *in vitro* and *in vivo* have identified a reduction in influenza virus replication (Jiang et al., 2011; Müller et al., 1994). Therefore, this SNP may reduce the silencing effect of gga‐miR‐1627‐3p binding, allowing greater IFNAR2 translation. Increased levels of IFNAR2 at the cell surface could lead to a more responsive activation of the type I interferon pathway upon influenza infection, resulting in a greater antiviral response.

Another example of a variant affecting a regulatory binding site was observed with the SNP at position Chr7:7933106, just upstream of *STAT1*, where a base substitution is predicted in a V‐MAF TF binding site. V‐MAF is an oncogene that was first identified in avian musculoaponeurotic fibrosarcoma virus; however, a V‐MAF protein homologue (C‐MAF) exists in chickens, sharing 99.7% identity. It is likely that the chicken C‐MAF will bind this same site, with binding affected in lines 6_1_, C, P2a and 0, altering *STAT1* expression in these lines. In humans and mice, C‐MAF has been shown to be involved in CD4 and CD8 T‐cell subset differentiation, function and interleukin production (Ho et al., 1996; Imbratta et al., 2020). Furthermore, research has indicated that *C‐MAF* expression is induced in a STAT1‐dependent fashion in CD4^+^CD25^−^ T cells. It is therefore possible that C‐MAF binding at STAT1 could act as a self‐upregulating positive feedback loop (Xu et al., 2009). *C‐MAF* overexpression has been linked to T‐cell lymphoma development in both mice and humans (Morito et al., 2006) and a key outcome in MDV pathogenesis is the development of T‐cell lymphomas (Ross, 1999). Interestingly, the SNP within the potential MAF binding site occurs in line 6_1_, which is known for its resistance to MDV, but is absent in its susceptible counterpart, line 7_2_. Considering C‐MAF’s involvement in cancer development and potential regulation by STAT1, this SNP may prevent C‐MAF binding upstream of *STAT1*. The lack of binding may mitigate carcinogenic effects that may be associated with upregulation of *STAT1*, leading to aberrant *C‐MAF* expression after MDV infection, and may explain the reduced pathogenesis and mortality by MDV seen in line 6_1_ (Mohd Isa et al., 2020).

Again, limitations on knowledge of TFBS and miRNA targets in chicken have to be considered when these predictions are made. Fifty‐two 3′UTR variants and 101 upstream variants were identified in this study. Given the incomplete knowledge of miRNA and TF binding sites it is possible that more of these variants than are currently identified affect binding sites and potentially contribute towards resistant and susceptible phenotypes.

In the current study, functional variants were mainly predicted from exonic, upstream and UTR regions. However, such an approach fails to consider the potential impact of 81.4% of the SNPs (1432 intronic SNPs) in the studied regions. SNPs in intronic regions could potentially impact splice sites, giving a different mRNA splice variant or affecting splicing efficiency, resulting in varying protein isoforms or expression levels which may affect phenotypes such as disease resistance (Cooper, 2010). SNPs outside of genes can also affect expression by impacting distal regulatory regions; TF binding at these sites may exist spatially close to promoter regions and influence transcription initiation despite being distant in terms of genomic coordinate (Wang et al., 2019).

Also requiring further research is the impact of heterozygous SNPs. As shown in Table S1, the vast majority of identified SNPs (78–95%) are homozygous. However, the smaller proportion of heterozygous SNPs may represent the accumulation of new mutations over time, reflecting selective advantage (as being homozygous may have detrimental consequence). Allele‐specific expression of genes is the result of differential expression occurring between the two parental alleles of one gene. This has implications in the context of this study as several SNPs were found to occur in only one allele and therefore may be upregulated or downregulated compared with the wild‐type allele. For example, the aforementioned missense SNP at position Chr30:182787 which results in a H215P substitution in the *TYK2* gene is present in the MDV‐susceptible line 7_2_ and absent from resistant line 6_1_. The JAK/STAT pathway has been previously found to contain SNPs exhibiting allele specific expression in response to MDV (Perumbakkam et al., 2013). It is possible that SNPs occurring in alleles that experience greater expression upon infection may have a significant effect on resistant or susceptible phenotypes. Considering the findings above, MDV infection may result in greater H215P‐TYK2 expression in line 7_2_. This amino acid substitution was predicted to be deleterious by provean and to affect protein function by snap2. The increased levels of this affected TYK2 may result in less ISG induction, leading to greater viral replication and more severe infection.

The ultimate goal will be to breed for a more resilient bird that can tolerate and recover from challenges without having lost productivity at the end of its rearing or laying period. Previous work indicated that selection for production traits has resulted in weakened immunity in commercial lines, but also suggested that selection for immunity was not detrimental to growth (Van der Most et al., 2011). Furthermore, a Genome Wide Association Study (GWAS) of indigenous African chickens found no correlation between production traits and immunity traits (Psifidi et al., 2016). Therefore, breeding schemes to introduce variants that result in higher innate immune responsiveness may not negatively impact production. Another economic advantage with using breeding schemes to introduce resilience is that this process does not require gene editing and will therefore not result in any consumer concerns over genetic modification; however, it will be a much slower process.

To enable breeding for more resilient birds we have to define the mechanisms of resistance, as a variant conferring resistance to one virus could lead to susceptibility against another. The observation that some SNPs appear to confer resistance in one line and susceptibility in another may be due to the level of interferon response. For example, some viruses may require a more robust response, which a particular SNP might yield, whilst with other viruses this more robust response may give rise to the observed ‘susceptibility’. For some viruses, a particular IFN may be detrimental and for others it will not because they have an ability to evade that response or to downregulate and block the induction of the IFN response. Hence breeding for resistance to virus A does not imply that it will also impart resistance to virus B. Our study indicated that the SNP at position Chr1:106603910 results in an R318K amino acid substitution in IL10Rβ. This SNP may contribute to IBDV resistance in line C whilst it may also contribute to MDV susceptibility in line 7_2_. This SNP may enhance the type III interferon pathway’s regulation of apoptosis (Li et al., 2008), which could therefore reduce bursal damage in IBDV‐infected birds owing to aberrant apoptosis (Cubas‐Gaona et al., 2018), but may contribute to the development of lymphoma in MDV infected birds (Xu et al., 2011). This example indicates why unravelling the mechanism for resistance is of importance, as breeding for variants at such loci may introduce susceptibility to other viruses outside the scope of this study.

It is only possible to postulate the mechanism by which the SNPs identified in this study function. Further research will be required to validate the effects of these variants on phenotype. It is interesting to note that none of the examined genes are located on chromosome 16, so any role in resistance/susceptibility is not due to co‐selection with the MHC during inbreeding of these lines. Furthermore, the current research is reductionist in considering only one SNP at a time; resistance and susceptibility are polygenic traits where effects may only be measurable in the presence of multiple SNPs working in concert with each other. The validation of candidate SNPs predicted in this work could be achieved by introducing variants *in vitro*, *in ovo* and *in vivo* and observing changes in antiviral responses, while also ensuring that responses to other pathogens are not negatively impacted. Our study further highlights the importance of the maintenance of rare, inbred chicken lines, given that they may already contain loci for resistance and once these have been identified a low‐density SNP array could be developed to easily inform breeding schemes.

## CONCLUSIONS

In the interferon pathway genes studied, 1760 SNPs were identified, of which 36 change an amino acid, 21 were predicted to affect mRNA secondary structure and three to affect TF/miRNA binding sites. Considering the research line phenotypes and the distribution of these SNPs, 78 instances could be related to resistance and 70 could be related to susceptibility to particular viruses. Further research will be required to validate these SNPs and examine the mechanisms involved in resistance or susceptibility. The current study has identified genomic variants in genes in the interferon pathways of different chicken research lines. This pathway is an integral component in innate immunity and can also contribute to the orchestration and development of the adaptive immune response. Not only will these findings help explain host innate immune responses and disease mechanisms used by these viruses and other micro‐organisms, but it may also assist in the identification of targets for selective breeding, drug design or vaccine improvement. Eventually, this will pave the way for breeding programmes that result in more robust commercial chicken lines.

## CONFLICT OF INTEREST

The authors declare that the research was conducted in the absence of any commercial or financial relationships that could be construed as a potential conflict of interest.

## Supporting information


Table S1

Table S2

Table S3

Table S4

## Data Availability

Genome sequence data are available in the European Nucleotide Archive under accession number PRJEB51681. All SNP information is provided in Table S4.
